# Identification and Expression Profiling of MicroRNAs in the Brain, Liver and Gonads of Marine Medaka (*Oryzias melastigma*) and in Response to Hypoxia

**DOI:** 10.1371/journal.pone.0110698

**Published:** 2014-10-28

**Authors:** Karen Lau, Keng Po Lai, Jessie Yun Juan Bao, Na Zhang, Anna Tse, Amy Tong, Jing Woei Li, Si Lok, Richard Yuen Chong Kong, Wing Yee Lui, Alice Wong, Rudolf Shiu Sun Wu

**Affiliations:** 1 School of Biological Sciences, Kadoorie Biological Sciences Building, The University of Hong Kong, Hong Kong SAR, China; 2 The State Key Laboratory in Marine Pollution, Hong Kong, Hong Kong SAR, China; 3 Genome Research Centre, The Hong Kong Jockey Club Building for Interdisciplinary Research, The University of Hong Kong, Pokfulam, Hong Kong SAR, China; 4 School of Life Sciences, Hong Kong Bioinformatics Centre, The Chinese University of Hong Kong, Hong Kong SAR, China; 5 Department of Biology and Chemistry, City University of Hong Kong, Kowloon, Hong Kong SAR, China; CNRS UMR7622 & University Paris 6 Pierre-et-Marie-Curie, France

## Abstract

The marine medaka (*Oryzias melastigma*) has been increasingly used as a fish model for detecting environmental stresses and chemical contaminants in the marine environment. Recent mammalian studies have shown that environmental stresses can alter the expression profiles of microRNAs (miRNAs), leading to transgenerational effects. Here, we use high-throughput Illumina RNA sequencing (RNA-Seq) for miRNA transcriptome analysis of brain, liver, and gonads from sexually mature male and female marine medaka. A total of 128,883,806 filtered sequence reads were generated from six small RNA libraries, identifying a total of 2,125,663 non-redundant sequences. These sequences were aligned and annotated to known animal miRNAs (miRBase) using the BLAST method. A total of 223 distinct miRNA types were identified, with the greatest number expressed in brain tissue. Our data suggested that 55 miRNA types from 34 families are common to all tested tissues, while some of the miRNAs are tissue-enriched or sex-enriched. Quantitative real-time PCR analysis further demonstrated that let-7a, miR-122, and miR-9-3p were downregulated in hypoxic female medaka, while miR-2184 was specifically upregulated in the testis of hypoxic male fish. This is the first study to identify miRNAs in *O. melastigma* using small RNA deep sequencing technology. Because miRNA expression is highly conserved between marine medaka and other vertebrates, marine medaka may serve as a good model for studies on the functional roles of miRNAs in hypoxia stress response and signaling in marine fish.

## Introduction

MicroRNAs (miRNAs) are small non-coding RNA molecules (∼22 nucleotides in length) encoded in the genome. They have emerged as one of the most important and abundant group of gene expression regulator in a wide range of species spanning from mammals to plants and viruses [1]. MiRNAs are first transcribed into primary miRNAs (pri-miRNAs) and then processed in the nucleus into precursor miRNAs (pre-miRNAs) with hairpin stem-loop structures. In animals, pre-miRNAs are then exported to the cytoplasm, where they are processed into mature miRNAs [2]. Mature miRNAs preferentially target 3′-untranslated regions (3′-UTRs) of mRNAs with which they share partial complementary sequence, leading to post-transcriptional gene silencing through translational repression. They can also lead to degradation of the target mRNA when complete sequence complementarity exists. Each miRNA may have multiple gene targets, and each gene target may also be regulated by more than one miRNA [3], [4].

Because of the important biological functions of miRNA, considerable efforts have been made to establish miRNA databases such as miRBase (http://www.mirbase.org), which detail all published miRNA sequences, annotations, and predicted gene targets [5]. The number of miRBase entries has increased exponentially since it was established in [6]. The latest version of miRBase comprises 30,424 distinct mature miRNA products from 206 species, including over 2,578 mature human miRNAs. Considerably fewer mature miRNAs have been reported in fish: 255 have been reported from zebrafish (*Danio rerio*), 108 from pufferfish (*Fugu rubripes*) and 146 from the Japanese freshwater medaka (*Oryzias latipes*) [5], [7]–[8]. Many of these fish miRNAs are conserved and have homologs in the human and mouse genomes [9]–[10].

Mammalian studies have shown that prenatal exposure to certain environmental stresses (e.g., hypoxia) and chemical contaminants (e.g., endocrine disrupting chemicals) can lead to alterations in phenotypes (e.g., delayed physical and sexual development, infertility, increased mortality and behavioral changes) in successive generations, despite the offspring have never been directly exposed to the stress and contaminants before [11], [12]. Such transgenerational effects may not be caused by alterations in DNA sequence but by changes in the epigenome. For example, changes may occur in DNA methylation, covalent modification of histones, or the activation or silencing of genes by miRNAs [13]. MiRNAs have been shown to modulate cellular differentiation and development, and various hypoxia-responsive miRNAs (HRMs) have been reported in human breast and colon cancers [14]–[16]. Although the functional roles of several HRMs have been extensively studied in mammals [17], [18], the biological roles of miRNAs in fish remain poorly understood. Small freshwater fish models (e.g. zebrafish and freshwater medaka) have been used extensively to identify and study biological responses to stresses in the freshwater environment [19], [20]. The marine medaka (which is very closely related to *O. latipes*) has been developed as a small fish model for similar studies in the marine environment [21]. However, the miRNA profile of this species is unknown.

New technologies such as high throughput RNA sequencing (RNA-Seq) have rapidly increased the rate of miRNA discovery. The high sensitivity of RNA-Seq allows for both the detection of species-specific, conserved miRNAs and the detection of weakly expressed miRNAs. MiRNA discovery has often relied on the mapping of small RNA sequence reads to a reference genome. Because a reference genome is currently not available for *O. melastigma*, predicting miRNAs in this species is difficult. However, with the development of alignment-type methods such as BLAST [22] for identifying homologs of known miRNAs, it has become possible to predict candidate miRNAs in the marine medaka. This is the first report on the identification and expression profiles of miRNAs in male and female *O. melastigma,* and also the differential responses of selected miRNAs to hypoxic stress. The results presented here will be invaluable for future transgenerational studies and risk assessment using this model marine fish species.

## Methods

### Medaka maintenance and RNA Isolation

All animal research procedures were approved by the Committee on the Use of Live Animals in Teaching and Research (CULATR, #2714-12) at the University of Hong Kong. The stock of marine medaka used in our experiment was obtained from Interocean Industries (Taiwan) and has been reared in our laboratory for over 10 generations. Marine medaka were maintained under optimal growth and breeding conditions as described in Kong *et al*, (5.8 mg O_2_ L^−1^, 28±2°C, pH 7.2 in a 14-h light: 10-h dark cycle) [21]. The fish were fed with hormone-free Aquatox Feed (Zeigler Bros. Inc. Gardners, PA) and live artemia. For hypoxia study, fifty juvenile male and fifty female marine medaka were reared under normoxia (5.8*+*0.2 mg O_2_/L) or hypoxia (1.5+0.2 mg O_2_/L) for three months. At 120 days post fertilization, the fish were anesthetized on an ice bath. Then, brain, liver, ovary and testis tissue were dissected from randomly selected male (*n* = 10 from normoxia and *n* = 10 from hypoxia) and female (*n* = 10 from normoxia and *n* = 10 from hypoxia) fish. Total RNA from pooled tissue samples was extracted using the mirVana miRNA isolation kit (Applied Biosystems) and treated with DNase (Ambion) to remove contaminating genomic DNA. RNA quality was assessed using the Agilent 2100 Bioanalyzer system and samples with a RNA Integrity Number (RIN) greater than 9 were used for small RNA library construction.

### Illumina small RNA library construction and sequencing

Six small RNA (cDNA) libraries were constructed (brain, liver and ovaries from female; brain, liver and testis from male), each prepared from 1 µg of total RNA. Small RNAs were gel purified to enrich for molecules less than 30 nucleotide in length, and Illumina adaptors (Integrated DNA technology) were ligated to their 5′ and 3′ ends to enable the construction of strand-specific cDNA libraries [23]. The ligation products were used as a template for cDNA synthesis using SuperScript II Reverse Transcriptase (Invitrogen), and an oligonucleotide primer with sequence complementary to the 3′ adapter was used to initiate cDNA synthesis (5′-CTCGGCATTCCTGCTGAACCGCTC-3′). cDNAs were amplified in 12 cycles with AccuPrime *Pfx* DNA Polymerase (Life Technologies) using PCR primer A (5′ -AATGATACGGCGACCACCGAGATCTACACTCTTTCCCTACACGACGCTCTTCCGATCT 3′) and PCR primer B (5′-CAAGCAGAAGACGGCATACGAGATCGGTCTCGGCATTCCTGCTGAACCGCTCTTCCGATCT 3′), and products were purified on Novex 8% TBE polyacrylamide gels (Invitrogen). Amplified DNA of 140–190 nt was extracted from the gel and purified using the QiaQuick Gel Extraction Kit (Qiagen). Sequencing was performed on the Illumina Genome Analyzer GAIIx. Sequences were extracted from image files using the Illumina pipeline, set at default parameters to yield sequence reads of 58 base lengths. Low-quality sequences, homopolymers and adaptor sequences were removed. The filtered reads were processed into different read lengths for further analysis.

### Illumina sequencing data analysis

Because the libraries were constructed strand-specifically, only reads in the sense orientation were considered in our analysis. The filtered reads were binned into non-redundant sets of unique small RNAs to tabulate the expression level profile for each library. The relative abundance of each unique miRNA was given as the number of tabulated reads per million filtered reads (RPM). Non-redundant miRNAs of between 20 and 23 bp having RPM≥6 were annotated using BLAST (Basic Local Alignment Search Tool) against miRBase version 17 [5], a database of known mature miRNA and mature star sequences. For unique species, only the highest-scoring alignments were considered for further analysis. Candidates were required to have at least 15 nucleotide matches to reference miRNAs with no more than 3 mismatches and to have an E-value lower than 0.06. In cases where a single reference miRNA from the database might match more than one sequence species from our *O. melastigma* small RNA library, the most abundant miRNA having the highest homology to the reference miRNA sequence was selected as the canonical candidate miRNA [24].

### qPCR analysis and identification of hypoxia-responsive miRNAs

TaqMan MicroRNA Assays (primer and probe sets PN: 4427975 and 4440886, Applied Biosystems) were used to detect and quantify five representative miRNA genes (let-7a, miR-9-3p, miR-27a, miR-122 and miR-2184) from the RNA tissue samples collected from male (*n* = 10 from normoxia and *n* = 10 from hypoxia) and female (*n* = 10 from normoxia and *n* = 10 from hypoxia) fish, according to the manufacturer’s protocol. U6 small nuclear RNA and 5S ribosomal RNA (5S rRNA) were used as an endogenous control for the analysis. All target sequences are shown in Table S1. cDNA was synthesized from total RNA using gene-specific primers. Reverse transcriptase reactions contained 10 ng of total RNA, 50 nM stem-loop RT primer, 1X RT buffer, 0.25 mM dNTPs, 3.3 U/µl MultiScribe reverse transcriptase, and 0.25 U/µl RNase inhibitor (TaqMan MicroRNA Reverse Transcription Kit, Applied Biosystems). The RT reactions were incubated in a C1000 Thermal Cycler (Bio-Rad) at 16°C for 30 min, then at 42°C for 30 min, at 85°C for 5 min and then held at 4°C. qRT-PCR was performed using the StepOnePlus Real-Time PCR system (Applied Biosystems). The 20 µl PCR included 1.3 µl RT product, 10 µl TaqMan Universal PCR Master Mix II (2X) without UNG (Applied Biosystems), 1 µl TaqMan Small RNA Assay, and 7.7 µl nuclease-free water. Reactions were incubated in a 96-well optical plate at 95°C for 10 min, followed by 40 cycles of 95°C for 15 sec and 60°C for 1 min. Reactions were run in triplicate and included a no-template control for each gene. Expression levels were determined based on the threshold cycle values (C_t_) of each target miRNA normalized to the 5S rRNA and U6 snRNA. Statistical significance was assessed using Wilcoxon–Mann–Whitney test.

### Availability of sequence data

The sequence data from this study have been submitted to the NCBI SRA (http://www.ncbi.nlm.nih.gov/sra) under the accession number SRP041922.

## Results

### Construction of small RNA libraries and Illumina sequencing

**Figure 1:**
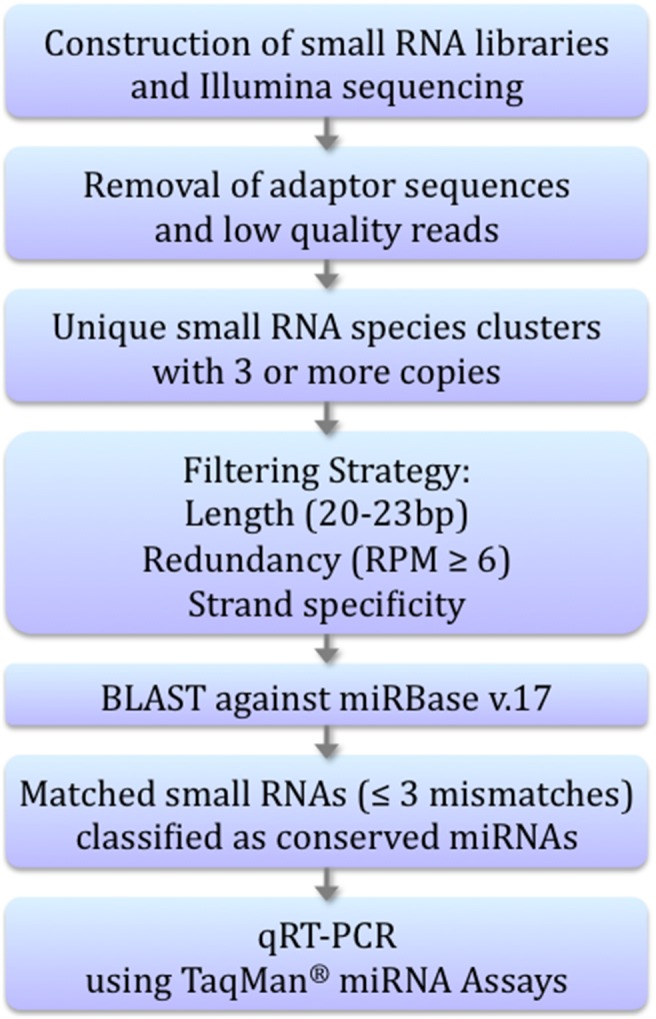
Schematic diagram of the workflow for *O. melastigma* miRNA discovery. Brain, liver and gonadal (ovary and testis) tissues of male and female marine medaka were submitted to small RNA libraries preparation and were sequenced using Illumina GAIIX. After the removal of low quality reads and filtering, high quality sequencing reads were blasted against miRBase v.17 to identify the conserved miRNAs of marine medaka.

To identify miRNA from marine medaka (*O. melastigma*), six small RNA libraries were generated and sequenced using the Illumina GAIIx. A schematic description of the workflow for *O. melastigma* miRNA discovery is shown in Figure 1. A total of 128,883,806 high quality filtered sequence reads were generated, and 2,125,663 non-redundant RNAs were identified. Between 18 and 25 million usable pre-processed reads were obtained for each library. The number of unique species following removal of redundant sequences is shown in Table 1.

**Table 1:** Statistics of pre-processed sequencing reads in six small RNA libraries of *O. melastigma*.

Small RNA Library	Number of Usable Reads	Total Number of Unique miRNA(All sizes)	Unique miRNA (20–23 bp)
Female brain	22,651,811	82,780	22,419
Female liver	18,898,991	54,580	8,763
Female ovary	22,890,085	745,265	55,506
Male brain	20,247,293	65,818	15,968
Male liver	18,660,890	68,024	10,463
Male testis	25,534,736	1,109,196	111,644

The length distributions of sequenced small RNAs in the six libraries were analyzed (Figure S1). In brain and liver, small RNAs of 21–23 nt in length were the most abundant species. However, in male and female gonads, the most abundant species are those between 26 and 28 nt. This distribution might reflect the presence of piRNAs in germ cells. It is consistent with the distribution that Houwing *et al* reported for zebrafish, in which ovary- and testis-specific piRNAs were detected [25]. The identification of piRNAs in ovaries and testis in the medaka will be assessed in future studies.

A stringent screening process for the most promising miRNA candidates was implemented, using sequence length (20–23 bp), expression of RPM≥6, and strand specificity as screening criteria. A total of 3,750 miRNAs were found to satisfy the criteria for potential miRNA (Table 2). The highest complexity was found in brain tissue, with 1,150 and 1,127 screened miRNAs detected in female and male brains, respectively. In contrast, liver, ovary and testis samples each yielded between 300 and 400 miRNAs.

**Table 2:** Annotation of canonical sequences based on known reference (miRBase).

Small RNA Library	Number of screened miRNA	Number of annotated canonical sequences	Number of annotated miRNA types
Female brain	1,150	243	198
Female liver	372	107	91
Female ovary	380	113	100
Male brain	1,127	241	195
Male liver	400	126	103
Male testis	321	89	83

The screened miRNAs satisfy four criteria: (1) sequencing length between 20 and 23 bp; (2) redundancy ≥6 RPM; and (3) strand specificity.

### Identification of miRNAs in marine medaka

To identify miRNAs in marine medaka tissues, all screened miRNAs were mapped against the current release of miRBase at the time of analysis [5]. Approximately 20–30% of the screened unique sequences from each library could be annotated as miRNA candidates based on homology to miRBase entries. Some canonical sequences from our libraries differed by only one or two bases from miRBase entries, indicating they are the likely marine medaka orthologs of those miRNAs. The annotated miRNA sequences for the six individual libraries are shown in Tables S2–S7. We also observed potential miRNA paralogs in our libraries: sequence variants related to canonical miRNA sequences. At this time, we cannot rule out the possibility that these are sequencing or amplification errors and not true paralogs encoded by related miRNA genes or arising by differential processing. However, the great sequencing depth of our study ensures a high level of accuracy. To simplify our dataset, potential paralogs were assigned the same miRNA number pending independent analysis. Using BLAST searches to reveal matches to known sequences, a total of 223 known miRNA types from 109 miRNA families were identified (Table S8), 183 of which were known miRNAs and 40 of which were known miRNAs* (all previously named star forms have been re-assigned the −5p/−3p nomenclature).

### Tissue- and sex-enriched miRNA expression in marine medaka

**Figure 2:**
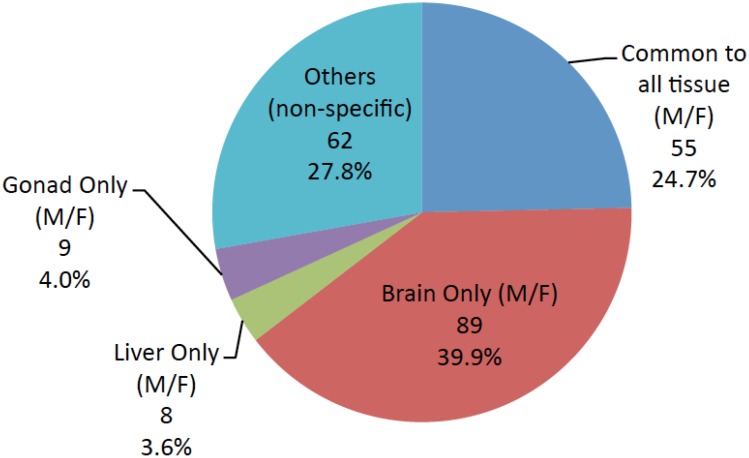
Pie chart of tissue-enriched miRNAs and conserved miRNAs common to all tissues in marine medaka. Diagram demonstrated brain-enriched, liver-enriched and gonad-enriched miRNAs of marine medaka. ‘Others’ are representative of miRNAs that are not tissue-enriched and not common to all male and female tissues.

**Figure 3:**
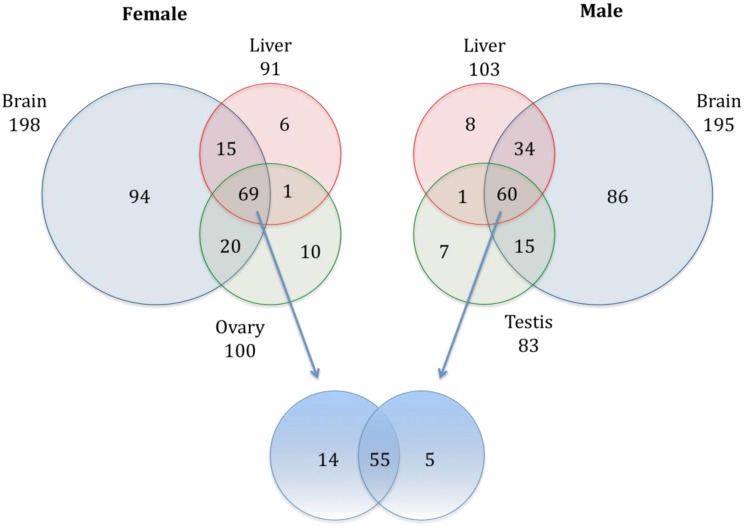
Venn diagrams of distribution of conserved miRNAs across tissues in female and male marine medaka. Diagram showed the distribution of conserved miRNAs in brain (grey), liver (red) and gonads (green) of marine medaka. Bottom diagram (blue) demonstrated the overlapped miRNAs between female and male medaka.

Tissue-enriched miRNAs in brain, ovary, testis and liver was elucidated. The highest numbers of genes were expressed in brain tissue (Table 3). In females, 198, 91, and 100 annotated miRNAs were identified in brain, liver and ovary, respectively. In males, 195, 103, and 83 annotated miRNAs were identified in brain, liver and testis, respectively. Comparison of expression profiles showed that 47.5% of identified miRNAs were tissue-enriched under the criteria used in this study: 39.9% of were brain-enriched, while only 3.6% were liver-enriched and 4% were gonad-enriched (Figure 2). The distribution of miRNAs across all three tissues in female and male medaka is shown in Figure 3; 69 miRNAs are found in female tissues and 60 miRNAs are found in male tissues, with only 55 miRNAs being common to all tissues in both males and females (24.7% of total miRNAs). A list of all commonly conserved miRNAs is shown in Table S9.

**Table 3:** Tissue- and sex-enriched miRNA expression in *O. melastigma*.

TissueType	Identified *O.melastigma* miRNA
Brain	let-7, let-7a-1-3p, let-7i-3p, miR-99-3p, miR-18a, miR-106, miR-10d, miR-124, miR-124a, miR-125b-1-3p, miR-129-1-3p, miR-129-3p, miR-129-5p, miR-130b, miR-132, miR-132-5p, miR-212, miR-135, miR-135b, miR-137, miR-137-5p, miR-137b, miR-138, miR-138-1-3p, miR-138-2-3p, miR-138b, miR-142-5p, miR-150, miR-153-5p, miR-153b, miR-22-5p, miR-22b, miR-23b-3p, miR-24-1-5p, miR-92a, miR-27b, miR-27b-5p, miR-365, miR-34a, miR-7-1-3p, miR-9a, miR-9a-3p, miR-9-3p, miR-181a-3p, miR-182, miR-183, miR-187, miR-190a-3p, miR-200b, miR-203b-5p, miR-216, miR-218, miR-218b, miR-219, miR-219-2-3p, miR-221-5p, miR-222a-5p, miR-301a, miR-301c, miR-375, miR-454, miR-455, miR-456, miR-458, miR-460b-3p, miR-489, miR-723, miR-724, miR-727, miR-727-5p, miR-728, miR-734, miR-737, miR-338, miR-2187, miR-2188-3p, miR-2188
Brain (Female only)	miR-20b, miR-124-5p, miR-133, miR-83, miR-184, miR-203a, miR-205a, miR-725, miR-962-3p
Brain (Male only)	let-7a-2-3p, miR-17-3p, miR-124-3p
Liver	miR-122, miR-122-3p, miR-148, miR-192, miR-199
Liver (Female only)	miR-4448
Liver (Male only)	miR-199b-3p, miR-749
Gonads	miR-196, miR-196a, miR-202, miR-202-5p
Ovary only	miR-27a, miR-1692
Testis only	miR-2895, miR-4682, miR-2184

**Figure 4:**
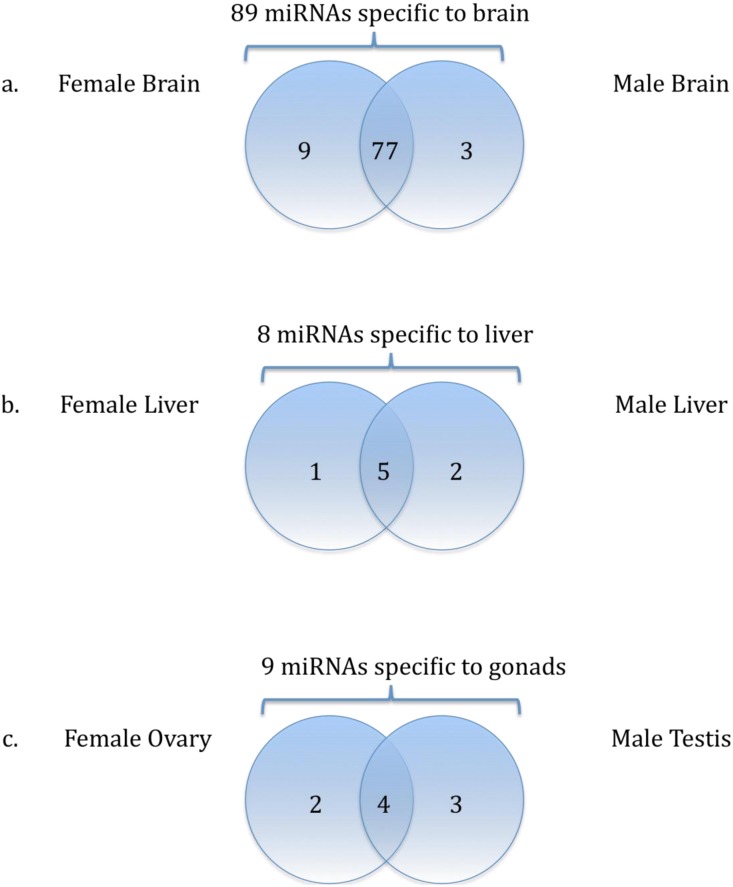
Venn diagrams of sex-dependent distribution of the tissue-enriched miRNAs. Diagram showed the overlapping of tissue-enriched miRNAs in a) brain, b) liver, and c) gonads of female and male medaka.

Further analysis showed that a small number of miRNA candidates are sex-enriched (Figure 4, Table 3). In the brain tissue, 89 miRNAs were identified, 9 of which (miR-20b, miR-83, miR-124-5p, miR-133, miR-184, miR-203a, miR-205a, miR-725, miR-962-3p) are only found in female brain and 3 (let-7a-2-3p, miR-17-3p, miR-124-3p) are enriched in male brain (Figure 4a). Of the 8 liver-enriched miRNAs, only 1 (miR-4448) is enriched in female liver and 2 (miR-749, miR-199b-3p) are only expressed in male liver (Figure 4b). We also observed 9 gonadal enriched miRNAs; 2 are only found in the female gonad (miR-27a, miR-1692) and 3 are enriched in the male gonad (miR-2184, miR-2895, and miR-4682) (Figure 4c).

### Highly expressed miRNAs in marine medaka tissues

Among the 223 orthologous miRNA types, miR-9a-3p was the most highly expressed in brain tissue, with RPM values of 23,080 and 26,939 in female and male, respectively (Tables S2–S3). Other highly expressed miRNAs in brain included let-7a, miR-29b and miR-153, each having RPM values greater than 6,000. In liver tissues, the most highly expressed miRNAs were miR-122 with RPM>1000 and miR-194 with RPM>6000 (Tables S4–S5). In gonads, the most highly expressed miRNA was let-7a, with RPM values of 3,563 in ovary and 2,351 in testis (Tables S6–S7). However, the expression levels of let-7a in gonads were fairly low compared to those in brain tissue: 13,722 RPM in females and 10,299 RPM in males. Other orthologous miRNAs that were abundantly expressed in ovary were miR-21, miR-26a, miR-26b, miR-216b, miR-202-5p and miR-143, and in testis miR-21, miR-29b, miR-216b, miR-202-5p, miR-2476, and miR-143.

### Interspecies comparison of miRNA expression in marine medaka tissues

The 223 identified miRNA types from 109 miRNA families were found to be homologous to miRNAs identified in 21 different species. Brain miRNAs showed a higher level of species conservation (21 species) than those identified in liver (15 species) and gonads (14 species). For a detailed species conservation analysis, *O. melastigma* miRNAs were compared with those identified in different model organisms including human (*Homo sapiens*), mouse (*Mus musculus*), nematode (*Caenorhabditis elegans*), fruit fly (*Drosophila melanogaster*) and four fish species: zebrafish (*Danio rerio*), tiger blowfish (*Fugu rubripes*), green-spotted pufferfish (*Tetraodon nigroviridis*) and Japanese freshwater medaka (*O. latipes*). BLAST searches revealed that the majority of identified *O. melastigma* miRNAs aligned well with human and zebrafish miRNA sequences (Table S10). A total of 144 of the identified *O. melastigma* miRNAs have previously been identified in zebrafish, and 92 have been identified in freshwater medaka. Only 3 *O. melastigma* miRNAs (Let-7, miR-1 and miR-124) were highly conserved across all model animals, 52 were conserved in all of the vertebrate models, and 31 were found to be teleost-specific (Table 4).

**Table 4:** Fish-enriched miRNAs identified in *O. melastigma*.

O. melastigma miRNA	Sequence	Species Conservation
let-7h	UGAGGUAGUAAGUUGUGUUGUU	dre, fru, tni
let-7j	UGAGGUAGUUGUUUGUACAGUU	dre, fru, tni
miR-10c	UACCCUGUAGAUCCGGAUUUGU	dre, fru, tni
miR-10d	UACCCUGUAGAACCGAAUGUGU	dre, fru, tni, ola
miR-16b	UAGCAGCACGUAAAUAUUGGAG	dre
miR-457a	AAGCAGCACAUCAUUACUGGUA	dre
miR-19d	UGUGCAAACCCAUGCAAAACUG	dre, ola
miR-22b	AAGCUGCCAGUUGAAGAGCUGU	dre, fru, tni
miR-27c	UUCACAGUGGUUAAGUUCUGC	dre, fru, tni, ola
miR-27e	UUCACAGUGGCUAAGUUCAGU	dre, fru, tni
miR-130c	CAGUGCAAUAUUAAAAGGGCAUU	dre, ola
miR-135c	UAUGGCUUUCUAUUCCUAUGUG	dre
miR-301c	CAGUGCAAUAGUAUUGUCAUA	dre
miR-456	CAGGCUGGUUAGAUGGUUGUCU	dre
miR-458	AUAGCUCUUUAAAUGGUACU	dre, fru, tni, ola
miR-460	CCUGCAUUGUACACACUGUGC	dre, fru, tni, ola
miR-462	UAACGGAACCCAUAAUGCAGCUG	dre, ola
miR-722	UUUUGCAGAAACGUUUCAGAUU	dre
miR-723	AGACAUCAGAAAAAUCUGUGCU	dre
miR-724	UUAAAGGGAAUUUGCGACUGUU	dre
miR-725	UUCAGUCAUUGUUUCUGGUCGU	dre
miR-727	UUGAGGCGAGUUGAAGACUUCA	dre
miR-728	AUACUAAGUAUACUACGUUUAC	dre
miR-730	UCCUCAUUGUGCAUGCUGUGUG	dre
miR-731	AAUGACACGUUUUCUCCCGGAUU	dre, ola
miR-734	UAAAUGCUGCAGAAUUGUGC	dre
miR-737	AAAUCAAAGCCUAAAGAAAAUA	dre
miR-1388	AUCUCAGGUUCGUCAGCCCAUG	dre, ola
miR-2184	AACAGUAAGAGUUUAUGUGCUG	dre
miR-2187	UUACAGGCUAUGCUAAUCUGU	dre
miR-2188	AAGGUCCAACCUCACAUGUCCU	dre

dre: *Danio rerio*; fru: *Fugu rubripes*; tni: *Tetraodon nigrovirdis*; ola: *Oryzias latipes*.

### qRT-PCR and stress response of marine medaka miRNAs

To validate miRNAs discovered during high-throughput sequencing, five representative miRNA candidates were selected for qRT-PCR analysis using RNA from the brain, liver and gonads of male and female *O. melastigma.* TaqMan MicroRNA Assays specific for mature miRNAs were performed with stem-loop RT primers. MiRNAs that were highly expressed in both sexes or that were tissue-enriched were chosen: let-7a (non-tissue enriched), miR-9-3p (brain-enriched), miR-122 (liver-enriched), miR-27a (ovary-enriched), and miR-2184 (testis-enriched). Consistent with our RNA sequencing data, qRT-PCR analysis (Figure S2) showed that let-7a was amplified in all of the male and female tissues examined, with the highest expression level observed in brain. Likewise, miR-9-3p was expressed only in the brain, while miR-122 was expressed only in the liver. As shown in our RNA-seq results, miR-2184 was most highly expressed in testis but was also found at extremely low levels in all other tissues. However, testicular expression of miR-2184 was significantly greater than that of all other tissues (*p*<0.05 for all). The highest level of miR-27a expression was observed in fish ovary. However, in contrast to the sequencing data, RT-PCR analysis indicated that miR-27a expression is not ovary-enriched. This discrepancy could be due to the presence of iso-miRNAs (multiple variants of miRNA genes) miR-27a, b, b-5p, c and e, which qRT-PCR may not be able to distinguish. Interestingly, RNA sequencing identified the presence of miR-27b and b-5p in fish brain only, while miR-27c expression was detected in all tissues and miR-27e in brain and gonads.

**Figure 5:**
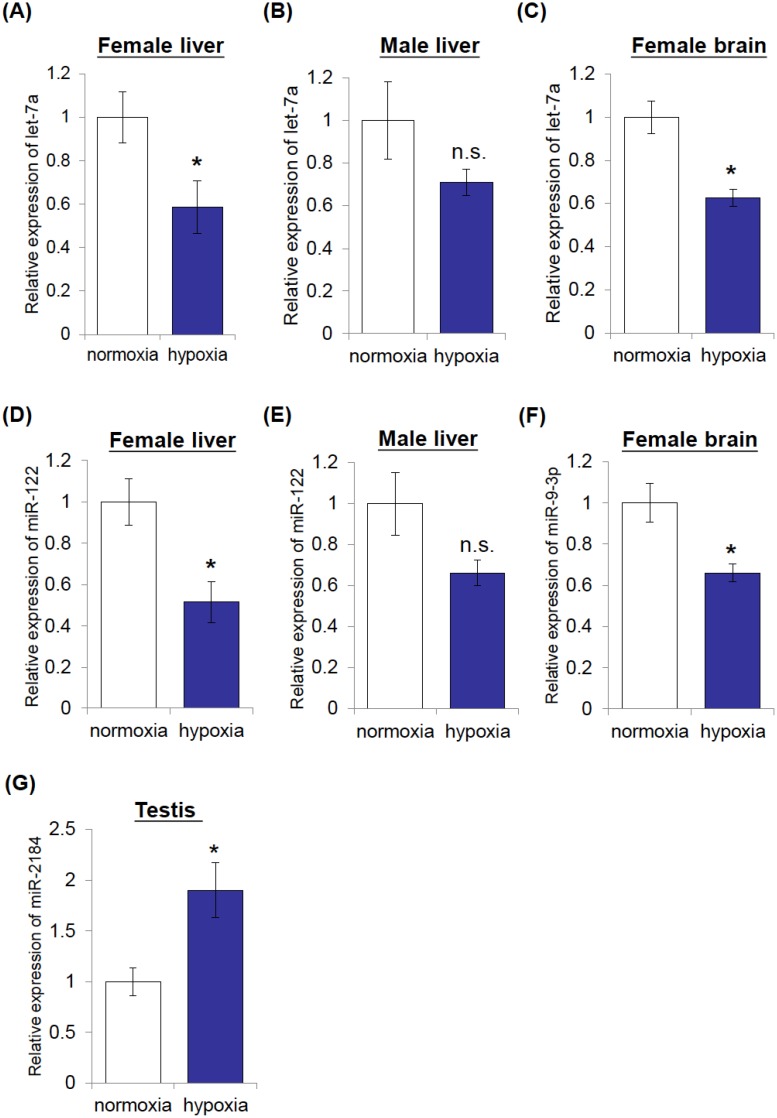
Identification of hypoxia-responsive miRNAs in marine medaka using qRT-PCR analysis. Female and male marine medaka were exposed to either hypoxia or normoxia for 3 months. The differentiation expressions of miRNAs in response to hypoxic stress were analyzed by quantitative real-time PCR. qRT-PCR analysis demonstrated the suppression of let-7a in female liver, male liver and female brain under hypoxia, respectively (A–C). Diagram showed the downregulation of miR-122 in female liver (D) and male liver (E). (F) Demonstrated the reduction of miR-9-3p in female brain. Diagram showed the upregulation of miR-2184 in testis of marine medaka (G). Data are presented as the means ± SEM (*p<0.05; n.s.: not significant).

Globally, hypoxia is an important environmental stressor affecting every area in the marine environment [26]. Experiments were therefore further carried out to test the response of five selected miRNA (let-7a, miR-9-3p, miR-27a, miR-122 and miR-2184) to hypoxic stress. qRT-PCR analysis was performed on the brain, liver and gonadal tissues of male and female medaka fish that have been exposed to normoxia or hypoxia for 3 months. Our results demonstrated a significant suppression of let-7a in hypoxic liver and brain tissues of female marine medaka as compared to normoxic tissues, and a similar reduction of let-7a was also found in male liver (Figure 5A–C). However, there is no significant change of let-7a in gonadal tissues and male brain of marine medaka (data not shown). Moreover, a profound inhibition of miR-122 was observed in hypoxic female and male liver (Figure 5D–E). A significant reduction of mi-9-3p was also demonstrated in hypoxic female brain, but not in male brain tissue (Figure 5F and data not shown). In gonadal tissues, a 2-fold induction of miR-2184 was found in testis in response to hypoxia (Figure 5G). However, there is no significant change of miR-27a in ovary under hypoxic exposure (data not shown). Taken together, our data indicated that hypoxia would deregulate the expression profile of tissue-enriched miRNAs in marine medaka, but the biological implications of these changes have yet to be elucidated.

## Discussion

The complete genome sequence of the *O. melastigma* has yet to be reported; thus, the established methods for novel miRNA discovery that involve mapping to the genome are not yet possible. The primary aim of the present study is to identify the known miRNAs in the organs along the brain-pituitary-gonad axis and the center of detoxification, liver in marine medaka. All of these organs are important for ecotoxicological studies. BLAST-based method was utilized to identify 223 distinct miRNA candidates in the brain, ovary, testis and liver of marine medaka based on homology to reported orthologous miRNAs. Using high-throughput sequencing, we identified unique miRNAs with expression levels ranging from 6 to nearly 27,000 RPM. Thus the methodology was highly sensitive and had sufficient dynamic range to detect miRNAs with both high and low expression levels. Our qRT-PCR analysis further confirmed that the identified *O. melastigma* miRNAs (let-7a, miR-9-3p, miR-122, miR-27a and miR-2184) are authentic miRNAs, which are differentially expressed by specific tissue and sex. The miRNAs were also highly tissue specific, with the majority brain-enriched. Many identified *O. melastigma* miRNAs in the present study are found to be highly conserved between species and taxa, and orthologous to miRNAs from as many as 21 other species, ranging from nematodes to humans. The majority of these miRNAs aligned well with miRNAs from humans and zebrafish and was also conserved in most vertebrates. Our analysis showed that 92% of *O. melastigma* miRNAs identified in the present study have orthologs previously found in zebrafish. Thus, species in the same taxonomic group appear to express similar miRNAs. The specific functions and gene targets of these teleost miRNAs are not well understood. Our qRT-PCR analysis further identified the hypoxia-responsive miRNAs: let-7a & miR-122 (female liver); let-7a & miR-9-3p (female brain) and miR2184 (testis) in hypoxia-exposed marine medaka.

In mammals, miRNAs are of great importance in the regulation of many fundamental biological processes such as cell proliferation, differentiation, apoptosis, signal transduction and organ development [27]–[29]. Several known classes of miRNAs have been predicted to regulate specific biological pathways. For example, the first known miRNAs, lin-4 and let-7, were found to play a major role in developmental timing [30], [31]. Highly expressed miRNAs are likely to have essential and broad regulatory functions. For example, mature let-7 regulates cell proliferation and differentiation and is also highly conserved across animal species [32]. Let-7 is one of the most highly expressed across all tissues in our data. Thirteen members of the let-7 family were identified in *O. melastigma*, 8 of which were common to the male and female tissue investigated (let-7a, let-7b, let-7d, let-7e, let-7g, let-7h, let-7i and let-7j). All let-7 members share a similar seed region which regulates the interaction between miRNA and its target genes, so it is generally believed that let-7 family imposes similar biological functions among different species. There is ample reports that demonstrated the importance of let-7 in different biological functions of brain, liver and gonads [33]–[35]. Sabrina *et al* reported that the introduction of let-7 caused the activation of Toll-like receptor 7, resulting in neurodegeneration in mouse’s brain [33]. Also, let-7 was found to control glucose homeostasis and insulin sensitivity in liver [34]. Furthermore, let-7 regulated ageing of testis stem cell niche in drosophila [35].

Other highly expressed miRNAs identified in *O. melastigma* include miR-9, miR-21, miR-29, miR-122, miR-124, miR-143 and miR-202-5p. The orthologous miRNAs identified in the present study have diverse annotated regulatory functions. The brain-enriched miR-9 and miR-124 are both known to have a crucial role in neurogenesis and neuronal development, particularly in the regulation of neural differentiation, proliferation and cell migration [36]–[38]. The expression of miR-122 was found to be liver-enriched. This is consistent with the results of previous studies and is consistent with its known important role in cholesterol and fatty acid metabolism [39]–[41]. Recently, let-7, miR-122 and miR-143 have been shown to have tumor suppressive activity, and de-regulated let-7 expression has been associated with cancer [14], [16], [41]. Some miRNAs have been implicated in the regulation of apoptosis including miR-21 and miR-29, which we found to be expressed in all medaka tissues. MiR-21 is thought to be anti-apoptotic and oncogenic, while miR-29 is thought to be pro-apoptotic, suppressing tumor growth in mice [42], [43]. We also observed gonad-enriched expression of miR-202-5p in medaka. This miRNA has been linked to the regulation of gonadal sex-differentiation and together with miR-21 appears to be estrogen-responsive [44], [45]. Thus, there is potential for highly expressed medaka miRNAs to respond to endocrine disruptors. However, the effects of such alterations are completely unknown.

Our data also demonstrated the suppression of let-7a in liver and brain tissues under hypoxic condition. This response to oxygen depletion is concordant to the previous finding that hypoxia caused reduction of let-7a in nasopharyngeal carcinoma [46]. Moreover, let-7 transgenic mice study indicated an impairment of glucose tolerance through the deregulation of insulin receptor (*Insr*) and insulin receptor substrate 2 (*Irs2*) in liver [34], suggesting that the hypoxia-mediated let-7a suppression may result in the dysfunction of medaka liver under hypoxia. But, it has been reported that let-7 can be induced by HIF-1α in response to hypoxia in vascular endothelial cell (HUVECs) [18]. Also, we found the suppression of miR-9-3p in brain tissue of hypoxic fish, which is contradictory to the findings in pulmonary artery smooth muscle cells where hypoxia caused the upregulation of miR-9 [47]. The opposite response of miRNAs to hypoxia treatment suggested that the differential expression of miRNAs in response to stresses may vary among different models. Moreover, we demonstrated the reduction of miR-122 in hypoxic liver tissues. It is similar to the finding in hypoxia-exposed Atlantic salmon [48]. The testis-enriched miRNA, miR-2184 is found to be upregulated in response to hypoxia. So far, there is no report to demonstrate the biological roles of miR-2184. But H-cadherin (CDH13), one of the predicted gene targets of miR-2184 reported in TargetScanFish, has been shown to be downregulated in testicular germ cell tumors and associated with differentiation of seminoma [49]. Thus, it would be interesting to study the functional roles of miR-2184-CDH13 pathway in testis in response to hypoxia.

Our previous report has demonstrated that hypoxia is an endocrine disruptor [50]. And, recent studies in mammals have suggested that endocrine-disrupting chemicals can modify the epigenome of the germ line via miRNAs, producing phenotypes in subsequent generations [13], [51]. The miRNA profiling and identification of hypoxia-responsive miRNAs of *O. melastigma* in this study serves as an important platform, not only for understanding fundamental biological processes but also provides new insight into the functional roles of miRNAs in responses to environmental stresses such as hypoxia. Conceivably, changes in miRNA profile and specific miRNAs may be employed as biomarkers for transgenerational effects of environmental stress such as hypoxia, which is likely to be an important emerging environmental problem.

## Supporting Information

Figure S1 **Length (nt) distribution of small RNAs in male and female brain, liver and gonads of** ***O. melastigma.*** Diagram showed distributions of small RNAs in female brain (upper left), male brain (upper right), female liver (middle left), male liver (middle right), female ovary (bottom left) and male testis (bottom right) of marine medaka.
(PDF)

Figure S2 **The qRT-PCR analysis of five representative miRNA candidates using TaqMan MicroRNA Assays.** (A) let-7a was amplified in all of the male and female tissues, (B) miR-9-3p was expressed in brain only, (C) miR-122 was expressed in liver only, (D) miR-2184 was most highly expressed in testis, and (E) miR-27a was most highly expressed in the ovary.
(PDF)

Table S1 Sequence information for all TaqMan MicroRNA Assays.
(XLSX)

Table S2 Annotated miRNA sequences and expression levels in female brain.
(XLSX)

Table S3 Annotated miRNA sequences and expression levels in male brain.
(XLSX)

Table S4 Annotated miRNA sequences and expression levels in female liver.
(XLSX)

Table S5 Annotated miRNA sequences and expression levels in male liver.
(XLSX)

Table S6 Annotated miRNA sequences and expression levels in female ovary.
(XLSX)

Table S7 Annotated miRNA sequences and expression levels in male testis.
(XLSX)

Table S8 Total conserved miRNAs identified in *O. melastigma*.
(XLSX)

Table S9 MiRNAs common to all *O. melastigma* tissues.
(XLSX)

Table S10 Highly conserved miRNAs identified in *O. melastigma*.
(XLSX)
