# Correction: Identification and Expression Profiling of MicroRNAs in the Brain, Liver and Gonads of Marine Medaka (Oryzias melastigma) and in Response to Hypoxia

**DOI:** 10.1371/journal.pone.0117543

**Published:** 2015-01-21

**Authors:** The PLOS ONE Staff

An error was introduced during the production process. One of the affiliations for the 9th author is not indicated. Richard Yuen Chong Kong is also affiliated with: Department of Biology and Chemistry, City University of Hong Kong, Kowloon, Hong Kong SAR, China. The publisher apologizes for the error.
