# Supplementary figures and images for: Identification and Expression Profiling of MicroRNAs in the Brain, Liver and Gonads of Marine Medaka (Oryzias melastigma) and in Response to Hypoxia
Source: PLoS One. 2014 Oct 28;9(10):e110698. doi: 10.1371/journal.pone.0110698 (PMC4211694; doi:10.1371/journal.pone.0110698)

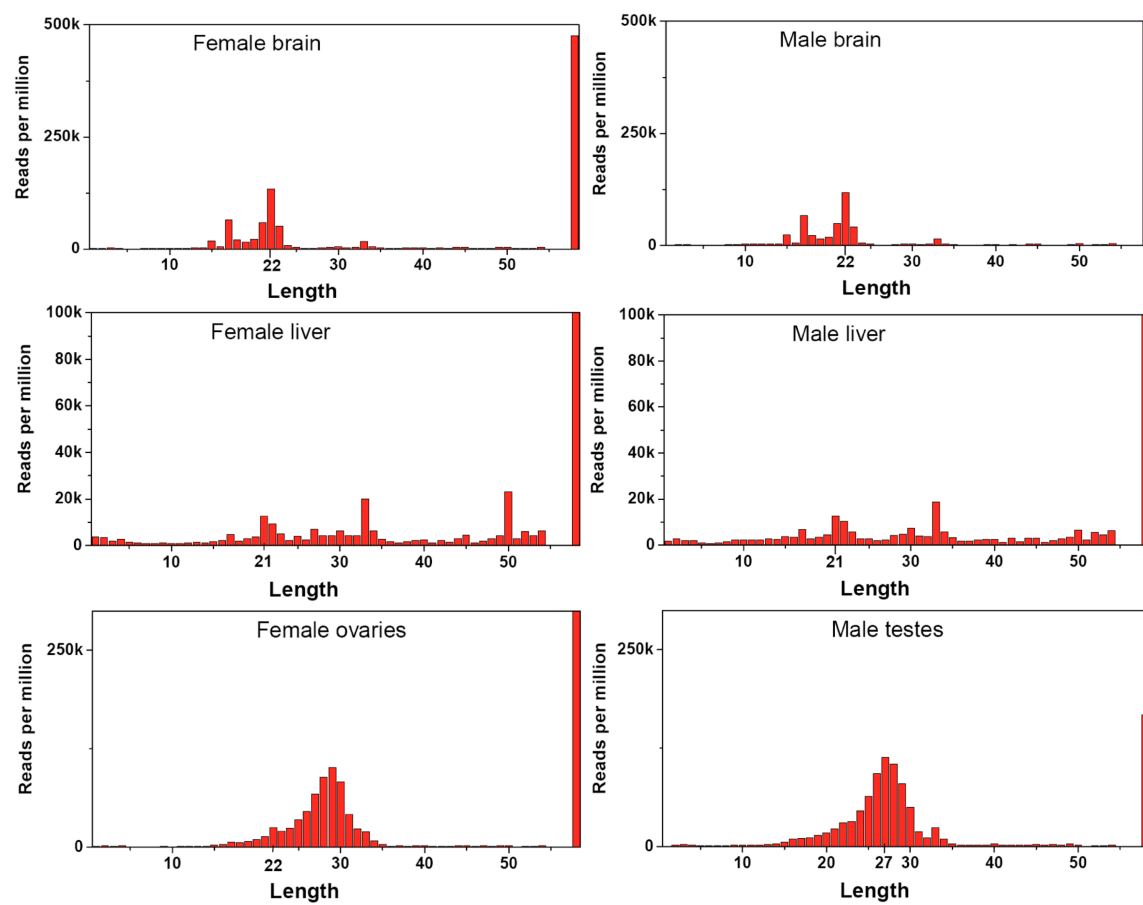

Figure 2

Supplement: Figure S1 — Length (nt) distribution of small RNAs in male and female brain, liver and gonads of O. melastigma. Diagram showed distributions of small RNAs in female brain (upper left), male brain (upper right), female liver (middle left), male liver (middle right), female ovary (bottom left) and male testis (bottom right) of marine medaka. (PDF) [file pone.0110698.s001.pdf]

a. Let-7a

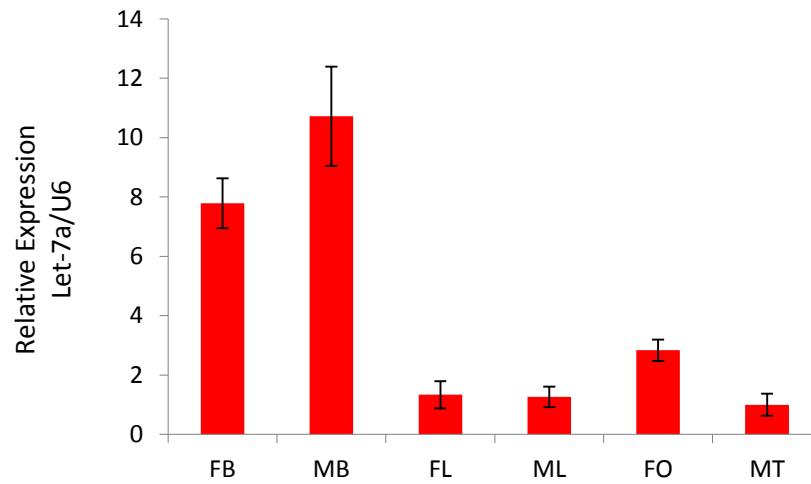

b. miR-9-3p (previously miR-9\*)

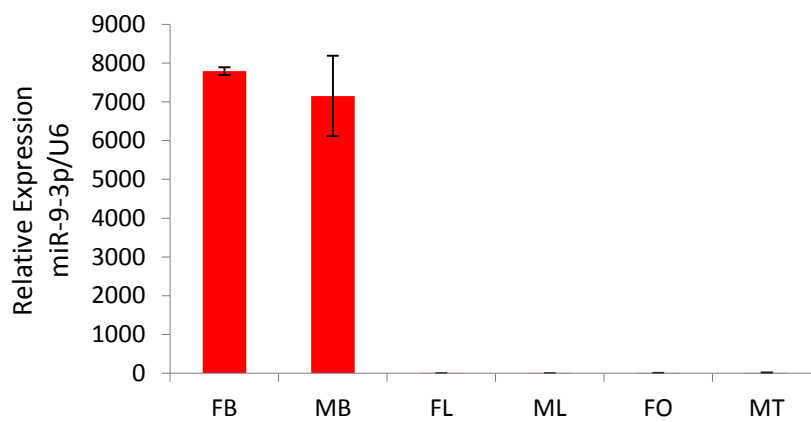

c. miR-122

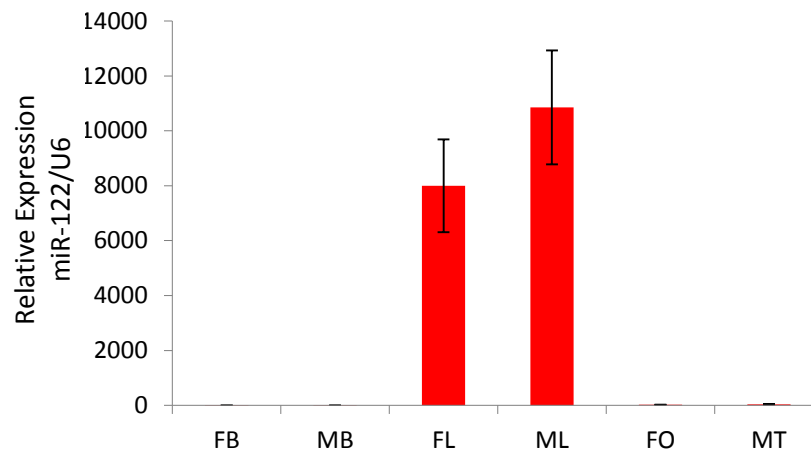

d. miR-2184

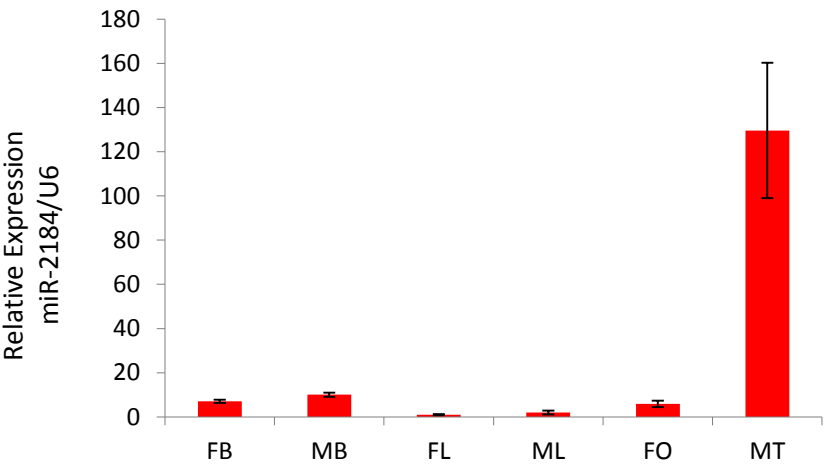

e. miR-27a

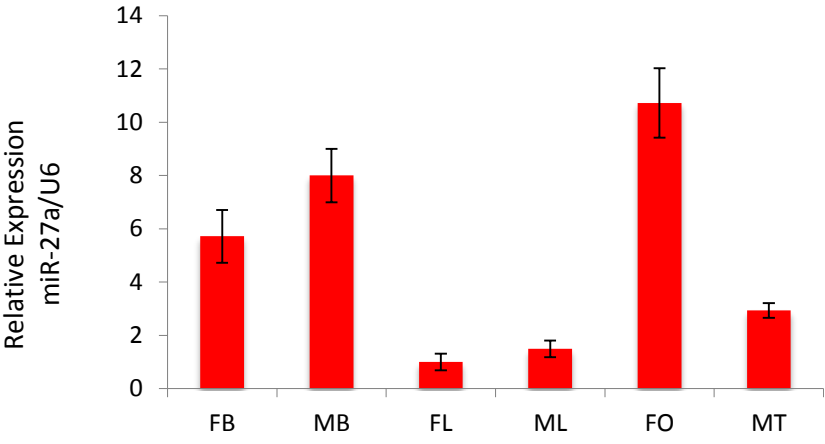

Supplement: Figure S2 — The qRT-PCR analysis of five representative miRNA candidates using TaqMan MicroRNA Assays. (A) let-7a was amplified in all of the male and female tissues, (B) miR-9-3p was expressed in brain only, (C) miR-122 was expressed in liver only, (D) miR-2184 was most highly expressed in testis, and (E) miR-27a was most highly expressed in the ovary. (PDF) [file pone.0110698.s002.pdf]
